# Progressive Visual Field Loss in the Setting of Severe Orbital Fat Atrophy

**DOI:** 10.7759/cureus.91711

**Published:** 2025-09-06

**Authors:** Konstantinos Kitsos-Kalyvianakis, Georgios Bontzos, Stefania Kyriazi, Efstathios T Detorakis, Eleni E Drakonaki

**Affiliations:** 1 Department of Ophthalmology, University of Crete, Heraklion, GRC; 2 Department of Ophthalmology, Korgialenio-Benakio General Hospital, Athens, GRC; 3 Department of Ophthalmology, General Hospital of Nikaia-Piraeus, Athens, GRC; 4 Department of Ophthalmology, University Hospital of Heraklion, Heraklion, GRC; 5 Department of Anatomy, University of Crete, Heraklion, GRC

**Keywords:** enophthalmos, glaucoma, optic nerve, orbital fat, prostaglandin

## Abstract

Prostaglandin analogues have a pivotal role in the management of glaucoma by effectively lowering intraocular pressure (IOP) and slowing disease progression. This study explores the complex case of an 81-year-old female with primary open-angle glaucoma, initially treated with travoprost. Despite controlled IOP, her visual fields deteriorated, particularly in the left eye. Investigations included sleep apnea tests, anemia blood tests, and orbital MRI, revealing optic nerve kinking. This case highlights the complex nature of glaucoma management, emphasizing the impact of mechanical stress on the optic nerve and the role of prostaglandin-induced orbital atrophy. The findings underscore the importance of personalized approaches, considering both pharmacological effects and individual anatomical variations.

## Introduction

Glaucoma is a leading cause of irreversible blindness, having a profound impact on affected individuals and a substantial burden on healthcare systems and society [1,2]. Understanding the risk factors associated with glaucoma is crucial due to their significant impact on the development and progression of the disease. Controlling intraocular pressure (IOP) stands as the primary treatment strategy for managing glaucoma due to its established correlation with disease progression.

Prostaglandin analogues, such as latanoprost, bimatoprost, and travoprost, are often utilized as a first-line therapy for reducing IOP in glaucoma patients due to their efficacy and relatively few side effects. However, some individuals may experience side effects such as ocular irritation, darkening of the iris or eyelid skin, or changes in periocular fat tissue.

It is important to note that the pathogenesis of glaucomatous optic nerve damage is multifactorial, involving both mechanical and vascular factors, and the interplay between these elements varies among individuals [3]. There is a significant body of evidence suggesting that mechanical stress of the optic nerve can cause glaucomatous damage [4,5]. In fact, the mechanical theory also applies in cases of acute IOP elevation where deformation of the lamina cribrosa occurs and axons passing this region are compromised [6]. The biomechanical properties of the optic nerve head and the peripapillary sclera have a significant role in the disease; thus, some individuals are more susceptible to glaucoma development [7].

In this context, we report a case of visual field worsening in a patient with significant orbital atrophy and optic nerve kinking. The functional deterioration was attributed to progressive orbital fat atrophy, which affected the intra-orbital course of the optic nerve and caused mechanical damage. Written informed consent was obtained from the patient.

## Case presentation

**Figure 1:**
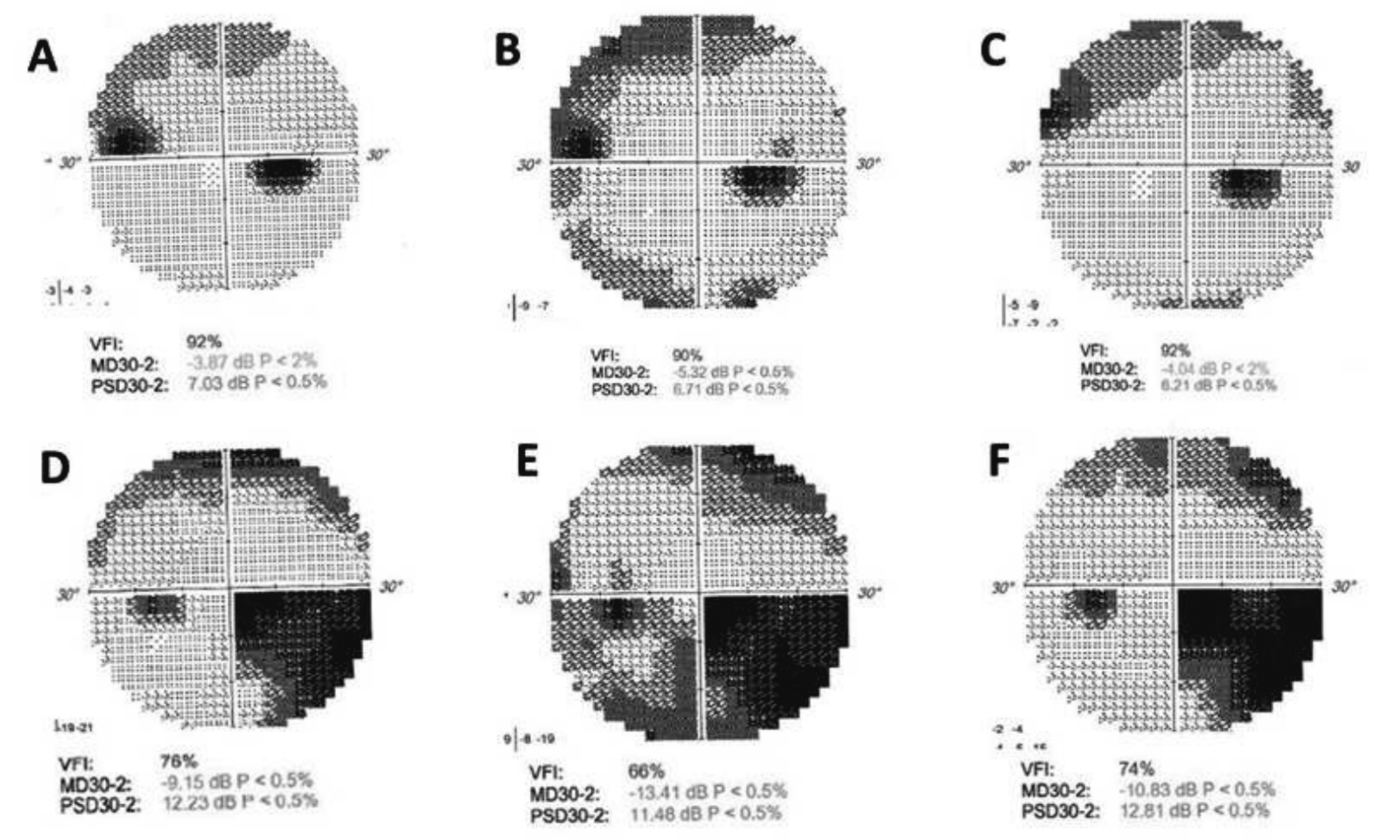
Visual fields in both eyes. Initial 30° automated visual field charts for OD (A-C) and OS (D-F), showing a superior nasal step and arcuate scotoma to the OD and an extensive inferior nasal step with superior arcuate scotoma to the OS. Respective visual field charts six months following the initiation of bimatoprost-timolol combination (B and E, respectively), with deterioration of findings in both eyes. Partial restoration of perimetric findings in both eyes six months following the discontinuation of bimatoprost-timolol (C and F, respectively). OD: right eye; OS: left eye.

An 81-year-old female patient was monitored for primary open-angle glaucoma (POAG) in both eyes, treated with travoprost for approximately 10 years. The patient had no history of hypertension, diabetes, or systemic vascular disease. Despite relatively low IOP, measured at 14 mmHg in the right eye (OD) and 16 mmHg in the left eye (OS) at her last visit, there was worsening in her visual fields, especially in the left eye (Figure 1). All visual field tests were considered reliable, with fixation losses <20% and false-positive/false-negative responses within acceptable limits. Central corneal pachymetry was 572 μm and 584 μm for OD and OS, respectively.

The patient underwent a detailed work-up for investigating low-pressure glaucoma, including blood pressure measurements, tests for sleep apnea, blood tests for anemia investigation, and orbital MRI.

**Figure 2:**
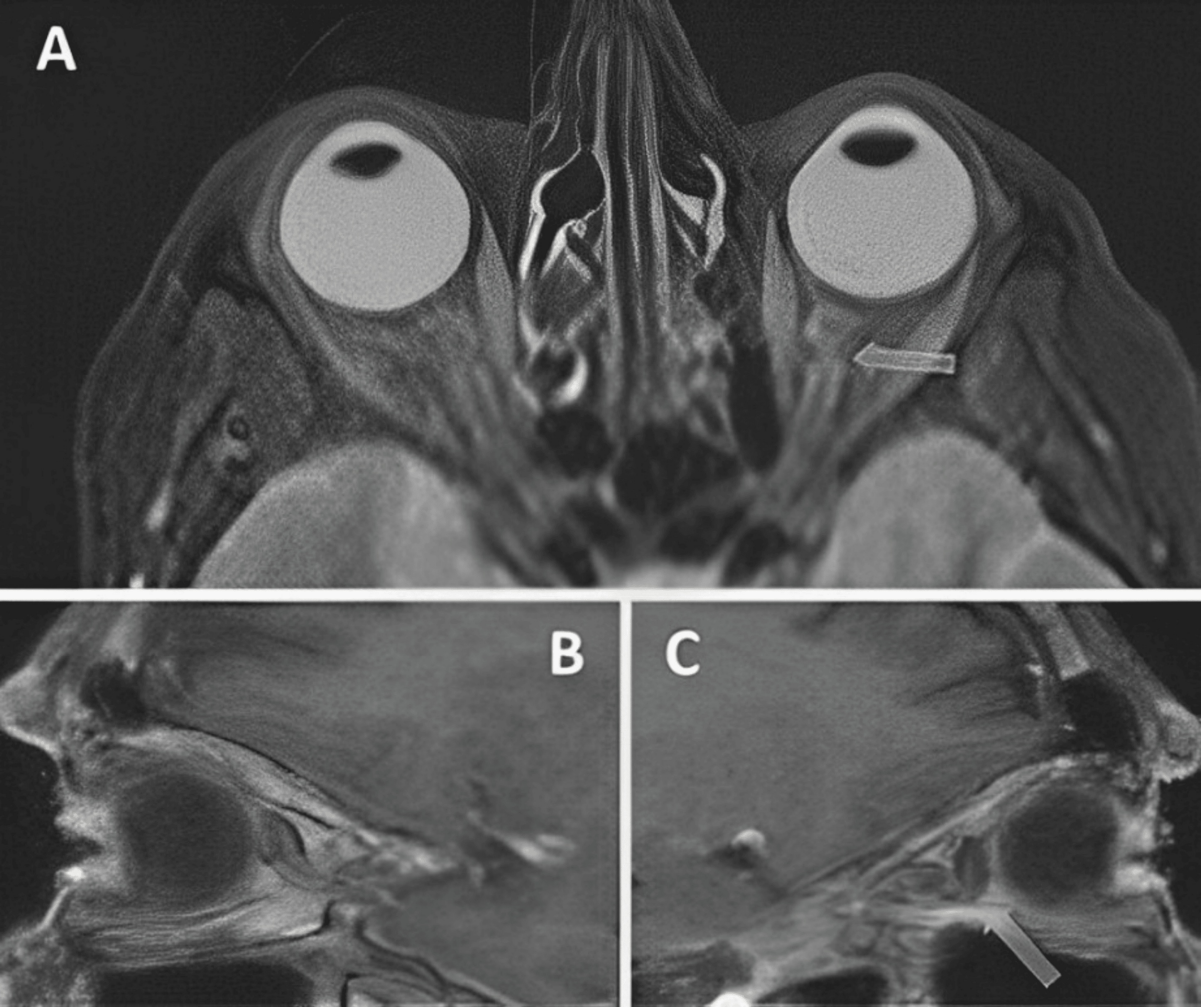
MRI showcasing the optic nerves. A: T2-oriented transverse MRI scan of the orbits, showing the kinking of the retrobulbar optic nerve in both eyes, more pronounced in the left eye (arrow). B, C: T1-oriented sagittal MRI scans of the right (B) and left (C) orbits, showing the kink in the retrobulbar course of the left optic nerve (arrow).

MRI revealed a tortuous course of both optic nerves, with significant kinking of the left nerve. On MRI, T1-weighted sequences were particularly useful for assessing orbital fat atrophy and enophthalmos due to their superior anatomical detail and fat contrast. T2-weighted sequences, on the other hand, better demonstrated the tortuosity and kinking of the optic nerve. Initially, the worsening of the visual fields was attributed to glaucoma progression, and treatment was switched to a fixed combination of bimatoprost-timolol, which the patient received for six months. Despite achieving lower IOP (10 mmHg in the right eye and 11 mmHg in the left), the visual fields continued to worsen, particularly on the left eye (Figure 2).

**Figure 3:**
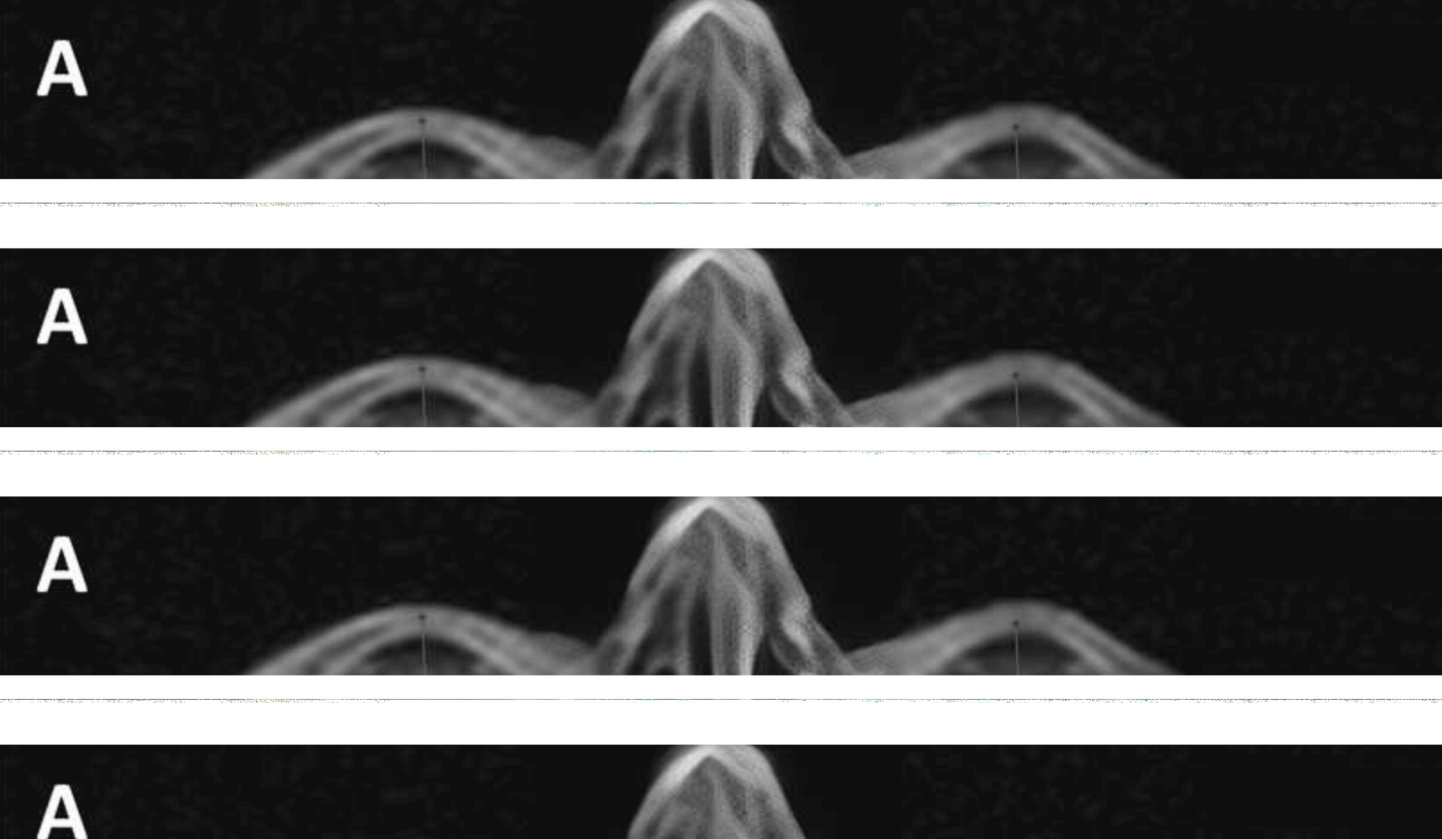
MRI scans showcasing enophthalmos. Enophthalmos, more pronounced on the left side, is evident by measurements from the intercanthal line to the orbital apex (16 mm for the OD and 13.5 mm for the OS) (A). Clinical presentation of the case, photographed from below, to show comparative left enophthalmos (blue line, B), and en-face, to show reduced size of the palpebral aperture to the OS (C). OD: right eye; OS: left eye.

The patient also developed enophthalmos, more pronounced especially on the left eye (Hertel exophthalmometry readings were 16 mm and 14 mm for OD and OS, respectively), which was attributed to orbital fat atrophy, associated with bimatoprost, and was confirmed by measurements taken in MRI scans from the intercanthal line to the corneal apex (Figure 3). Enophthalmos was diagnosed based on Hertel exophthalmometry and MRI measurements. In clinical practice, enophthalmos is generally defined as a posterior displacement of the globe greater than 2 mm compared to the fellow eye or relative to normative population values (typically 16-18 mm from the lateral orbital rim to the corneal apex).

By taking into account the tortuous retro-bulbar optic nerve course, especially for the OS, we assumed that the deterioration of the visual fields, despite the low IOP levels and the absence of other predisposing factors, could be attributed to a compressive effect exerted by the retro-placed eyeballs, especially on the left side, causing further kinking in the optic nerve course and potentially affecting its function. After six months of treatment with bimatoprost-timolol, visual fields continued to worsen, particularly in the left eye.

It was considered that enophthalmos due to bimatoprost-induced fat atrophy in the orbit could cause functional impairment of the optic nerve, particularly if it was associated with severe kinking. Given the MRI findings and worsening enophthalmos, therapy was changed to brimonidine-brinzolamide, which the patient continued for six months, with relative stabilization of visual fields.

## Discussion

In this case, we investigated a patient with progressive functional loss of vision, as shown in her visual field tests, in an otherwise controlled glaucoma. The progression continued despite medication changes and further IOP reduction. We considered this situation as a potential side effect of orbital atrophy, probably due to the prostaglandin drops that were used. Despite the chronic use of the medication, the patient probably reached a critical point where the optic nerve was mechanically damaged due to its severe kinking and lack of orbital fat support. Follow-up neuroimaging after discontinuation of prostaglandin therapy was not performed; therefore, potential reversal of optic nerve kinking could not be objectively documented. However, the partial recovery of visual field sensitivity and the clinical improvement of enophthalmos suggest a functional component related to orbital fat atrophy.

Prostaglandin analogues are often used as first-line treatment for POAG and are usually well tolerated [8-10]. However, patients may experience side effects such as topical hyperemia, increased iris pigmentation, trichomegaly, and orbital fat atrophy [11,12]. Prostaglandin-associated peri-orbitopathy, first described in 2004 [13], can be observed clinically as the absence of dermatochalasis, a deep upper eyelid crease suggestive of levator dehiscence, ptosis, and enophthalmos of approximately 2 mm. These signs are typically more severe in patients receiving travoprost and bimatoprost [14]. The underlying mechanism involves activation of the prostaglandin F receptor, which inhibits preadipocyte differentiation and downregulates fatty acid-binding protein expression, reducing uptake of free fatty acids and triglyceride synthesis [15,16]. Although prostaglandins are administered topically, part of the drug diffuses through conjunctival and scleral tissues and accumulates in periocular and orbital fat, including the intraconal compartment, via local penetration and vascular or lymphatic spread. Consequently, long-term use may lead to diffuse orbital fat atrophy, with clinical manifestations such as enophthalmos and, in severe cases like ours, alteration of the mechanical course of the optic nerve. Interestingly, discontinuation of prostaglandins often results in partial reversal of these changes [17].

Pressure of the optic nerve, especially in the region of the lamina cribrosa, is strongly associated with the development of glaucoma. External stress of the optic nerve can lead to scleral canal expansion and anterior displacement of the lamina cribrosa, which can impair axonal transport and damage the retinal ganglion cells [18,19]. It has been hypothesized that, in biomechanical terms, the movement of the eye globe involves mechanical strain on the tissues supporting the orbit. Typically, in healthy individuals, when the optic nerve and its sheath are stretched in certain eye movements, they are more flexible, allowing them to absorb strain and reduce eye globe deformation. In contrast, for people with POAG, the optic nerve and sheath are thought to be abnormally stiff. This stiffness means they stretch less during these movements, potentially transferring strain to the more vulnerable areas near the optic nerve head [20].

A potential limitation of this case is the possibility of fluctuation or learning effects in automated perimetry, which can sometimes mimic disease progression. However, we believe this was unlikely, as the patient had long-standing experience with perimetric testing, with stable and reliable indices in prior examinations. Furthermore, the visual field changes followed a consistent pattern across sequential tests and correlated with anatomical findings on MRI and clinical evidence of enophthalmos, reducing the likelihood that the observed deterioration was due to test variability alone. The reliability indices of the perimetric examinations were within acceptable limits, which further supports the validity of the observed progression.

## Conclusions

To our knowledge, no prior cases have been reported linking prostaglandin analogue use to optic nerve kinking secondary to orbital fat atrophy. Most studies on prostaglandin-associated peri-orbitopathy describe more common features, such as enophthalmos, upper lid sulcus deepening, and ptosis. Our report, therefore, adds a novel manifestation to this spectrum. From a physiological standpoint, prostaglandin analogues, though administered topically, can reach deeper orbital tissues via conjunctival and scleral diffusion, as well as vascular and lymphatic spread. Activation of prostaglandin F receptors in orbital adipocytes inhibits preadipocyte differentiation, downregulates fatty acid-binding proteins, and promotes lipolysis, ultimately leading to diffuse orbital fat atrophy. This mechanism explains how topically applied agents can affect not only superficial periocular fat but also intraconal fat compartments, with potential to alter the position of the globe and the course of the optic nerve.

Clinical features from this case can help us further understand the pathophysiology and risk factors involved in glaucoma. Continuous efforts are made to develop innovative diagnostic tools and novel pharmacological agents to mitigate disease progression, reaffirming the significance of addressing the multifactorial pathogenesis in glaucoma. Although this is a single case report, the high prevalence of glaucoma and the associated widespread use of bimatoprost in the population imply that the possibility of optic nerve function impairment in anatomically predisposed orbits should be taken into consideration in glaucoma management. Therefore, this possibility could be further investigated with larger prospective studies.
